# The Effect of Poor Social Support on Depression among HIV/AIDS Patients in Ethiopia: A Systematic Review and Meta-Analysis

**DOI:** 10.1155/2020/6633686

**Published:** 2020-12-09

**Authors:** Adisu Birhanu Weldesenbet, Sewnet Adem Kebede, Biruk Shalmeno Tusa

**Affiliations:** ^1^Department of Epidemiology and Biostatistics, College of Health and Medical Sciences, Haramaya University, Haramaya, Ethiopia; ^2^Department of Epidemiology and Biostatistics, Institute of Public Health, College of Medicine and Health Sciences, University of Gondar, Gondar, Ethiopia

## Abstract

**Background:**

Low- and middle-income countries of which Ethiopia is one bears the high burden of depression among human immune deficiency virus and acquired immune deficiency syndrome (HIV/AIDS) patients. Several factors have been identified as being associated with increased depression among HIV/AIDS patients including poor social support. However, studies examining the effect of poor social support on depression among HIV/AIDS patients in Ethiopia have had inconsistent findings. This systematic review and meta-analysis is therefore aimed at estimating the pooled effect of poor social support on depression among HIV/AIDS patients in Ethiopia.

**Methods:**

All relevant articles published prior to July 1, 2020, were retrieved from scientific databases: PubMed, Scopus, and Google Scholar systematically. The identified studies reporting the association of depression and poor social support among HIV patients in Ethiopia were included. *I*^2^ tests were used to assess the heterogeneity of the studies. Subgroup analysis was done based on tools to determine how pooled estimates of depression vary across tools. The pooled estimate of association between poor social support and depression was reported.

**Results:**

The aggregated meta-analysis revealed a higher odds of depression among patients with poor social support than those who had strong social support (OR: 2.31, 95% CI: 1.69, 2.93). The pooled prevalence of depression among HIV/AIDS patients in Ethiopia was 38.93% (95%: CI: 32.01, 45.84); (*I*^2^ = 94.44%, *p* ≤ 0.001). The subgroup analysis was performed based on tools, and the result showed that the highest pooled prevalence (44.42%) was among primary studies that used the Hospital Anxiety and Depression Scale (HADS) tool.

**Conclusions:**

Human immune deficiency virus and acquired immune deficiency syndrome (HIV/AIDS) patients with poor social support were more likely to develop depression. The pooled prevalence of depression among HIV/AIDS patient was high in Ethiopia. The highest prevalence of depression was observed among studies that used HADS to screen depression. Therefore, we recommend integration of mental health and psychosocial support services into the HIV/AIDS care. Prevention of HIV/AIDS-related stigma for people with HIV/AIDS is also needed to reduce the impact of poor social support.

## 1. Background

Globally, 36.9 million people are living with human immune deficiency virus and acquired immune deficiency syndrome (HIV/AIDS) and more than half of them are in Africa. The greatest burden of the disease is concentrated in developing countries. Mental health is highly intertwined with communicable diseases such as HIV. In Ethiopia, reports indicate that the overall prevalence of HIV/AIDS is 1.1% among all ages and 2.1% among adults. Mental disorders like depression put people at a higher risk for contracting HIV, and among those living with HIV, its associated stigmatization can lead to poor mental health outcomes [1–3].

Depression is a common mental disorder that presents with depressed mood, loss of interest or pleasure, decreased energy, feelings of guilt or low self-worth, disturbed sleep or appetite, and poor concentration. Approximately, 350 million people are currently living with depression. It is the fourth leading cause of disability worldwide. Its lifetime prevalence was one in five women and one in ten men [4, 5].

Depression is the most common mental disorder among HIV/AIDS patients with prevalence rates of about 60%. Studies found that people living with HIV had twice the risk for depression than those who were at risk for HIV. Depression and HIV/AIDS are estimated to be the world's two leading causes of disability by 2030 [6, 7]. Low- and middle-income countries bear high burden of depression among HIV/AIDS patients. The prevalence of depression among people living with HIV (PLWHIV) in sub-Saharan Africa was 9 to 32% [8]. In Ethiopia, it ranges from 7.3 to 73. 3% [9, 10].

Mental health problems are associated with increased risk of HIV infection and interfere with their treatment, and, conversely, some mental disorders occur as a direct result of HIV infection. Depression affects a person's ability to follow treatment for HIV/AIDS. It is associated with poor adherence to antiretroviral therapy (ART) leading to immunological failure and may independently increase HIV progression [11–13].

Depression influences health-seeking behavior and outcome of HIV/AIDS treatment and increases progress and burden of disease and the risk of mortality and morbidity for HIV patients. The symptoms of depressive disorders decrease adherence to HIV ART leading to the drug-resistant virus, and untreated depressive disorders decrease immune status [14–16].

Decreased social support within the context of HIV/AIDS is related to increased depression because of various factors such as educational disability and food insecurity. Low social support could result in poor adherence to medication, and as a result, poor adherence leads to immune suppression which finally leads to depression. Poor social support may lead to social isolation, which can be responsible for depression. Social isolation by HIV patients itself reduces social support that can result in a negative impact on their physical and mental well-being. This is also supported by the fact that these patients might prefer to avoid seeking help from others, and in addition, social stigma towards them could increase their isolation and loneliness [17–19].

In order to overcome the negative impact of depression among HIV/AIDS patients, evidence on association of social support with depression is of paramount importance. While the relationship between poor social support and depression is fairly well-established in high-income settings, fewer studies have investigated the association in low-resource setting like Ethiopia. Studies that have examined the association between poor social support and depression among PLWHA in Ethiopia in particular have presented inconsistent results, with some finding strong positive associations [20, 21] and others finding negative associations [22].

Therefore, this systematic review and meta-analysis is aimed at estimating the pooled effects of poor social support on depression among HIV/AIDS patients in Ethiopia. This finding will help decision makers and other stakeholders working on mental health to reduce the magnitude of depression and the associated disability among HIV/AIDS patients by implementing effective interventions.

## 2. Methods

### 2.1. The Protocol and Registration

We conducted this systematic review and meta-analysis based on the Preferred Reporting Items for Systematic Review and Meta-Analysis (PRISMA) statement guideline [23]. The protocol for this review was registered on International Prospective Register of Systematic Reviews (PROSPERO) with reference number CRD42020201157.

### 2.2. Search Strategies

Comprehensive search strategy was made on depressive symptoms and associated factors among HIV/AIDS patients to identify all relevant studies. A systematic literature search for the relevant papers was made in PubMed, ScienceDirect, and Google Scholar. The search was restricted to papers published prior to August 1, 2020, in Ethiopia and published in English. Population, Intervention, Comparison, and Outcome (PICO) format was used to search the relevant studies. “Social support”, “Depression”, Depressive symptoms, “Effect”, “HIV”, “AIDS”, and “Ethiopia” were combination of relevant keywords used.

### 2.3. Eligibility Criteria

#### 2.3.1. Inclusion Criteria

All observational studies conducted on the prevalence of depression; studies that assessed the association of social support with depression in Ethiopia; studies published and accessible before August 1, 2020; articles written in English; and citations with abstract and/or full text were eligible for current systematic review and meta-analysis.

#### 2.3.2. Exclusion Criteria

Articles which were not fully accessed because of the inability to assess the quality of articles in the absence of full text, duplicate reports, systematic reviews and meta-analyses, qualitative studies, and inconsistent outcome measures were excluded from the review.

### 2.4. Study Selection

The selection of studies from electronic databases was made based on titles and abstracts, and in cases when a definite decision could not be made based on the title and/or abstract alone, the full paper was obtained for detailed assessment of the inclusion criteria. Two authors (BST, SAK) screened and evaluated studies independently. The other author (ABW) independently evaluated the quality of the studies against the checklist, and if any discrepancy arises in decision process, it was resolved through discussion or through asking a third reviewer if consensus could not be reached. Then, the full text of the studies were further evaluated based on objectives, methods, and study populations.

### 2.5. Outcome Measurement

The outcome variable of interest was depression and defined as presence of depressed mood, loss of interest or pleasure, decreased energy, feelings of guilt or low self-worth, disturbed sleep or appetite, and poor concentration. In the included studies, social support is measured using the Oslo 3-item social support scale with individuals who were scored less than 9 regarded as having poor social support, adolescent social support rating scale with those scoring 1-2.9 considered having poor social support, and Social Support Questionnaire-6 (SSQ-6) with patients who scored below mean having low social support.

Perceived HIV stigma was measured using an 11- and 12-item perceived HIV stigma scale consisting of questions about disclosures, and patients who scored greater than or equal to mean were classified as internally stigmatized. We determine the association between depression and poor social support in the form of the odds ratio.

### 2.6. Data Extraction Process

A data extraction template was used to extract necessary data from the articles with format containing the title of the study, author's name, study designs, year of publication, sample size, study population, and effect size by two independent groups of reviewers. Data from selected articles were extracted using a data extraction template and presented through Microsoft Word.

### 2.7. Quality Appraisal

The Joanna Briggs Institute (JBI) quality appraisal criteria established for analytical cross-sectional studies were used. The quality of the findings of the included articles was critically evaluated using the quality assessment tool for observational studies [24]. The two groups of authors (SAK and BST) and ABW independently evaluated the quality of the studies. The reviewers compared their quality appraisal scores and resolved any discrepancy before calculating the final appraisal score. Articles with an appraisal score of ≥6 out of 10 scales were considered high quality and were considered eligible.

### 2.8. Statistical Analysis

Standard error of proportion for all included studies was computed using the binomial distribution formula. Heterogeneity among reported prevalence was assessed by computing *p* values of *I*^2^-statistics. For meta-analysis with significant heterogeneity, random-effects model was used and subgroup analysis was performed. The subgroup analysis was conducted based on tools to investigate how depression varies across different categories of tools used for outcome measurement.

Publication bias was assessed by Egger's tests at 5% significant level. Odds ratio (OR) with 95% CI was used to examine the association between poor social support and depression. The pooled prevalence rate of depression was expressed as a point estimate and 95% CI. The prevalence from each study was weighed by the sample size. All data manipulation and statistical analysis were performed using Stata software version 16.

### 2.9. Ethical Approval

Since the review was concerned with the research articles, there was no need for ethical approval and/or additional consent from participants.

## 3. Results

### 3.1. Search Result

**Figure 1:**
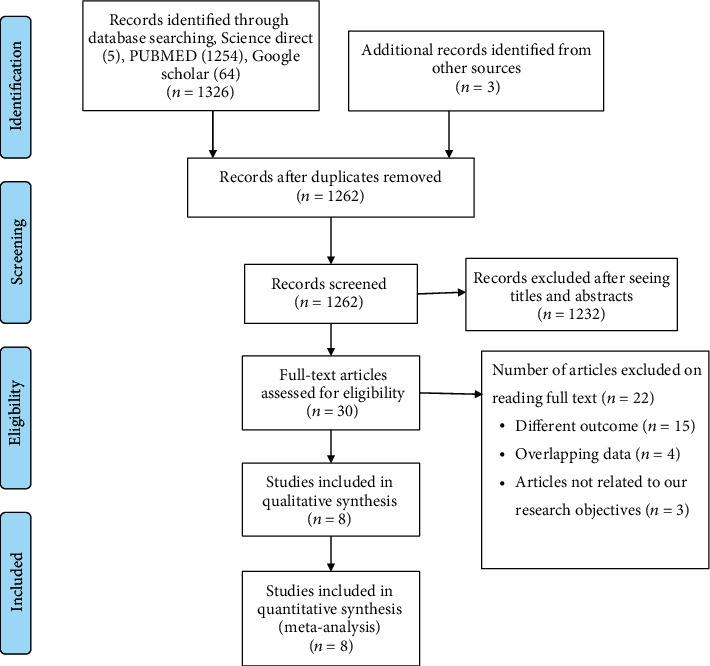
PRISMA flow chart showing the selection of primary studies.

Initially, a total of 1326 articles were retrieved using scientific databases (PubMed = 1254, Science Direct = 5, Google Scholar = 64). Additional 3 articles were also searched from Google. After removing duplications, 1262 articles were considered eligible for title and abstract evaluation. Accordingly, 1232 articles were excluded and the remaining 30 articles were considered for further full-text evaluation. After the full-text reading, 22 articles were further excluded due to differences in the outcome of interests, study objectives, and overlapping of the data. Finally, 8 papers were found as eligible to be included in this systematic review and meta-analysis (Figure 1).

### 3.2. Characteristics of Included Studies

The studies included in this systematic review and meta-analysis were all cross-sectional. They had a total of sample of 3287 adults living with HIV/AIDs in Ethiopia and were conducted from 2016 to 2020 in different regions of the country. The study sample sizes ranged from 340 reported from a study in Southern Nations Nationalities, and People (SNNP) [25] to 507 from a study in Addis Ababa [26] participants. Of the eight studies included in the final analysis, three were conducted in Addis Ababa [21, 26, 27], two studies were from the SNNPR region [25, 28], two studies were conducted in Amhara regions [20, 22], and the remaining one study was from Oromia region [29].

The response rates of the studies ranged from 93.5% [22] to 100% [21, 27]. The mean age of the patients included in this systematic review and meta-analysis ranged from 18.6 ± 3.024 years [26] to 38 ± 10.228 [28]. Regarding assessment tools of depressive symptoms, five studies used the Patient Health Questionnaire-9 (PHQ-9) [22, 25, 27–29], two studies used Hospital Anxiety and Depression Scale (HADS) [20, 21], and the other one study used Beck Depression Inventory-II (BDI-II) [26] (Table 1).

### 3.3. Meta-Analysis of Depression in Ethiopia

**Figure 2:**
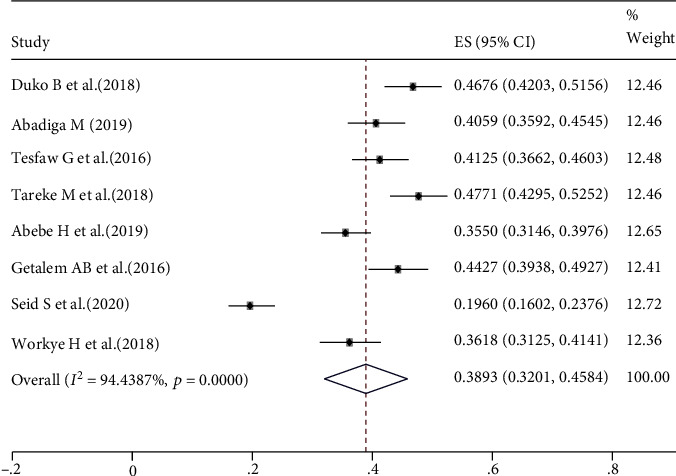
The pooled estimated prevalence of depression among HIV/AIDS patients in Ethiopia, 2020.

The prevalence of depression in this systematic review and meta-analysis ranged from 20% in Dessie [22] to 48.6% from studies in Hawassa and Bahir Dar [20, 28]. The pooled prevalence of depression among HIV/AIDS patients in Ethiopia was 38.93% (95%: CI: 32.01%, 45.84%) (Figure 2).

### 3.4. Subgroup Analysis

**Figure 3:**
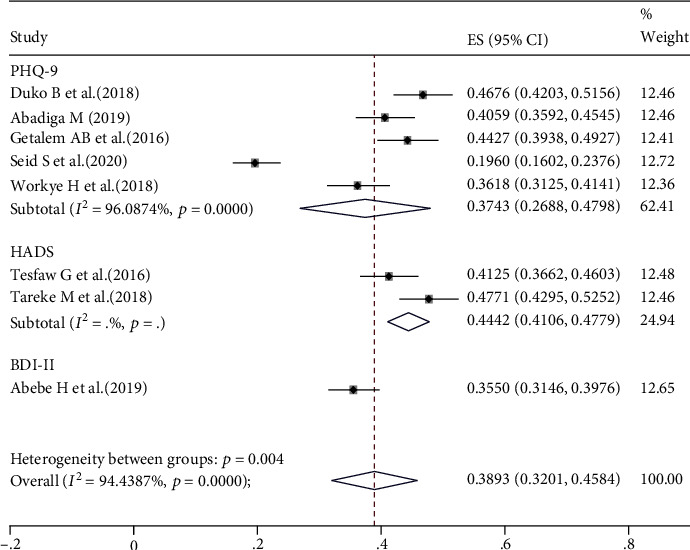
The pooled estimated prevalence of depression among HIV/AIDS patients in Ethiopia based on tools, 2020.

There was a significant heterogeneity (*I*^2^ = 100%, *p* ≤ 0.001) across the included studies. Therefore, we performed subgroup analysis based on the tool used for outcome measurement to estimate the pooled prevalence of depression in Ethiopia. The pooled prevalence of depression was 44.42% (95% CI 37.65%, 52.15%) among two studies that used HADS and 37.43% (95% CI 28.74%, 48.14%) for five studies that used PHQ-9. One study indicated that the prevalence of depression among HIV/AIDS patients was 35.50% (95% CI: 35.46, 35.54) using BDI-II (Figure 3).

### 3.5. The Impact of Perceived Stigma on Depression

The highest odds ratio (OR) for impact of poor social support on depression was 9.97 reported from a study conducted in Oromia region [29] and can be interpreted as the odds of having depressive symptoms among adults living with HIV who had poor social support were 9.97 times higher than those who had strong social support. The smallest odds ratio for poor social support (2.02) was reported from a study conducted in Addis Ababa [21] showing that the odds of depressive symptoms is 2.02 times more likely among those with poor social support as compared to those with strong social support.

**Figure 4:**
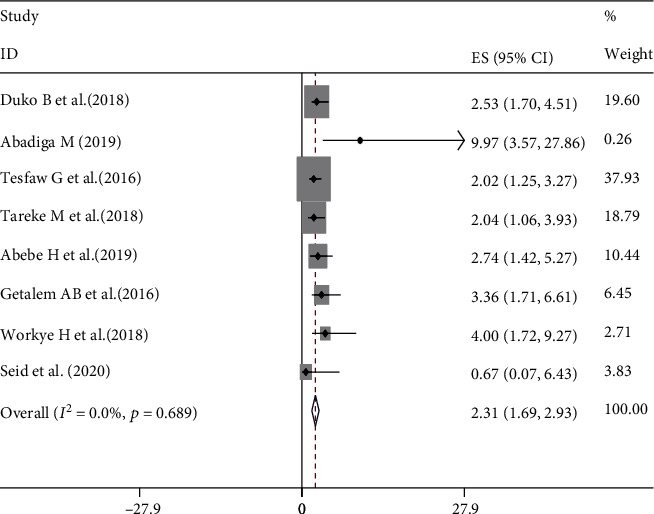
Forest plot of pooled OR between poor social support and depression among HIV/AIDS patients in Ethiopia.

In seven of eight included studies [20, 21, 25–29], there was a significant association between poor social support and depression. The aggregated meta-analysis using eight studies revealed HIV/AIDS patients with poor social support had 2.31 times higher odds of developing depression as compared to those who had strong social support (OR: 2.31, 95% CI: 1.69, 2.93) (Figure 4).

### 3.6. Publication Bias Results

Funnel plot and Egger's test was used to evaluate the presence of publication bias. Each dot in the funnel plot represents a single study. The *y*-axis is usually the standard error of odds ratio. Larger studies with higher power are placed towards the top whereas lower powered studies are placed towards the bottom. The *x*-axis shows the odds ratio.

**Figure 5:**
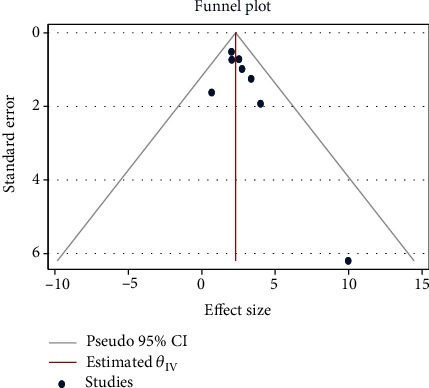
A funnel plot for assessing publication bias.

For the current review as depicted in Figure 5, the plot is symmetric indicating there is no evidence of publication bias or small study effect in our study (*p* value from Egger's test = 0.065). However, in this interpretation, it should be put into consideration that the funnel plot and Egger's test are less reliable when the number of studies is less than 10.

## 4. Discussion

This systemic review and meta-analysis attempted to estimate the pooled effect of poor social support on depression among HIV/AIDS patients in Ethiopia. The analysis revealed that poor social support had a statistically significant effect on depression among HIV/AIDS patients. The odds of having depression among adults living with HIV who had poor social support were 31% higher than those who had strong social support. The positive relationship between poor social support and depression in the current study is consistent with a finding from a systematic review in Africa [30]. The reason might be due to the fact that patients who did not share their problems with other people had stress and social isolation by HIV patients itself reduces social support that can result in a negative impact on their physical and mental well-being. The fact that these patients might prefer to avoid seeking help from others and in addition, social stigma towards them could increase their isolation and loneliness might also contribute to positive relationship between poor social support and depression [18, 19].

The pooled prevalence of depression among HIV/AIDS patients in the current study was 38.93%. This finding is in line with the prevalence of depression among HIV/AIDS reported from low- and middle-income countries (12 to 78%) [31]. The current finding was higher than the study in sub-Saharan Africa (9 to 32%), and the possible reason might be due to the difference in sample size, methodology, and tools used to assess depression.

We conducted subgroup analysis based on tools, and the highest prevalence was observed among primary studies that used HADS (44.42%), and it was 37.43% among primary studies that used the PHQ-9 tool. The disparity might be due to the difference in the number of primary studies. Only two primary studies used HADS as a tool to assess depression whereas five studies used PHQ-9, and this may increase precision of the pooled estimate for depression.

The strength of this review is it was conducted with rigorous adherence to the PRISMA checklist which improves its quality for the readers. The main limitation of this meta-analysis is it may be lacking national representativeness since primary studies were found only from four administrative regions, namely, Addis Ababa, Amhara region, Oromia region, and SNNP region; this could bias the estimated prevalence of depression for the entire Ethiopian context.

## 5. Conclusions

The pooled prevalence of depression among HIV/AIDS patient was high in Ethiopia. The highest prevalence of depression was observed among studies that used HADS. Human immune deficiency and acquired immune deficiency syndrome patients with poor social support were more likely to develop depression. Therefore, we recommend the integration of mental health and psychosocial support services into the HIV/AIDS care. The prevention of HIV/AIDS-related stigma for people with HIV/AIDS is also needed to reduce the impact of poor social support.

## Figures and Tables

**Table 1:** Descriptive characteristics of 8 studies included in the systematic review and meta-analysis of effect of poor social support on depression among HIV patients in Ethiopia, 2020.

Authors (reference)	Publication year	Study area/region	Study design	Tools	Response rate	Mean age (±SD)	Sample size	Prevalence
Duko et al. [28]	2018	SNNP	Cross-sectional	PHQ-9	96%	38 ± 10.228	417	48.6%
Abadiga [29]	2019	Oromia	Cross-sectional	PHQ-9	97%	25.6 ± 9.45	404	41.7%
Tesfaw et al. [21]	2016	Addis Ababa	Cross-sectional	HADS	100%	37.44 ± 10.07	417	41.2%
Tareke et al. [20]	2018	Amhara	Cross-sectional	HADS	98%	36.9 + 10.5	415	48.6%
Abebe et al. [26]	2019	Addis Ababa	Cross-sectional	BDI-II	94%	18.6 ± 3.024	507	35.50%
Getalem and Emnet [27]	2016	Addis Ababa	Cross-sectional	PHQ-9	100%	38	384	44.40%
Seid et al. [22]	2020	Amhara	Cross-sectional	PHQ-9	93.5%	38 (IQR = 10)	403	20%
Workye et al. [25]	2018	SNNP	Cross-sectional	PHQ-9	96.5%	37.6 (*R* = 50)	340	37.50%

SD: standard deviation; SNNP: Southern Nation, Nationalities, and People; PHQ-9: Patient Health Questionnaire-9; HADS: Hospital Anxiety and Depression Scale; BDI-II: Beck Depression Inventory-II; IQR: interquartile range; R: range.

## Data Availability

All necessary information were included with in the manuscript.

## Abbreviations

AIDS: Acquired immune deficiency syndrome
ART: Antiretroviral therapy
BDI-II: Beck Depression Inventory-II
CI: Confidence interval
HADS: Hospital Anxiety and Depression Scale
HIV: Human immune deficiency virus
JBI: Joanna Briggs Institute
OR: Odd ratios
PHQ-9: Patient Health Questionnaire-9
PICO: Population intervention comparison and outcome
PLWHA: People living with HIV/AIDS
PRISMA: Preferred Reporting Items for Systematic Review and Meta-Analysis
PROSPERO: Prospective Register of Systematic Reviews
SSQ-6: Social Support Questionnaire-6
SNNP: Southern Nation, Nationalities, and People.
